# A Network Pharmacology-Based Study on the Hepatoprotective Effect of *Fructus Schisandrae*

**DOI:** 10.3390/molecules22101617

**Published:** 2017-09-28

**Authors:** Ming Hong, Yongsheng Zhang, Sha Li, Hor Yue Tan, Ning Wang, Shuzhen Mu, Xiaojiang Hao, Yibin Feng

**Affiliations:** 1 School of Chinese Medicine, Li Ka Shing Faculty of Medicine, The University of Hong Kong, 10 Sassoon Road, Pokfulam, Hong Kong, China; hong1986@connect.hku.hk (M.H.); alex.yszhang@gmail.com (Y.Z.); u3003781@connect.hku.hk (S.L.); hyhtan@hku.hk (H.Y.T.); ckwang@hku.hk (N.W.); 2 Institute of Clinical Pharmacology, Guangzhou University of Chinese Medicine, 12 Jichang Road, Guangzhou 510405, China; 3 Zhejiang Chinese Medical University, 548 Binwen Road, Hangzhou 310053, China; 4 The Key Laboratory of Chemistry for Natural Products of Guizhou Province and Chinese Academy of Sciences, Guiyang 55500, China; muzi0558@126.com (S.M.); haoxj@mail.kib.ac.cn (X.H.); 5 State Key Laboratory of Phytochemistry and Plant Resources in West China, Kunming Institute of Botany, Chinese Academy of Sciences, Kunming 650000, China

**Keywords:** Wuweizi, network pharmacology, hepatoprotective effect

## Abstract

*Fructus schisandrae* (Wuweizi in Chinese), a common traditional Chinese herbal medicine, has been used for centuries to treat chronic liver disease. The therapeutic efficacy of Wuweizi has also been validated in clinical practice. In this study, molecular docking and network analysis were carried out to explore the hepatoprotective mechanism of Wuweizi as an effective therapeutic approach to treat liver disease. Multiple active compounds of Wuweizi were docked with 44 protein targets related with viral hepatitis, fatty liver, liver fibrosis, cirrhosis, and liver cancer. A compound–target network was constructed through network pharmacology analysis, predicting the relationships of active ingredients to the targets. Our results demonstrated that schisantherin, schisandrin B, schisandrol B, kadsurin, Wuweizisu C, Gomisin A, Gomisin G, and angeloylgomisin may target with 21 intracellular proteins associated with liver diseases, especially with fatty liver disease. The CYP2E1, PPARα, and AMPK genes and their related pathway may play a pivotal role in the hepatoprotective effects of Wuweizi. The network pharmacology strategy used provides a forceful tool for searching the action mechanism of traditional herbal medicines and novel bioactive ingredients.

## 1. Introduction

Liver diseases, including viral hepatitis, fatty liver, liver fibrosis, cirrhosis, and liver cancer, are major diseases threatening human health and are the leading cause of deaths worldwide [1]. Although there has been remarkable progress in the treatment of liver diseases over the last several decades, most of the therapies still do not yield satisfactory outcomes in patients [2]. In view of the scarce treatment options and significant adverse effects caused by conventional chemical agents, novel prophylactic and therapeutic agents against liver disease are urgently needed [3].

*Fructus schisandrae* (Wuweizi in Chinese), the fruit of *Schisandra chinensis* (Turcz.) Baill., is a traditional herbal medicine, which is believed to be liver tonic in China, Japan, and Russia [4]. According to the theories of traditional Chinese medicine (TCM), Wuweizi can be used for treating Liver and Kidney Deficiency of Yin or Yang syndrome [5]. Modern pharmacological study showed that Wuweizi might exhibit various therapeutic effects, such as hepatoprotection, anti-inflammation, and antioxidant properties [4,6,7,8]. Recent studies demonstrated that the extract from Wuweizi could alleviate hepatic cholesterol and triglyceride levels, and retard the development of fatty liver in rodents given high-fat diets [9]. Wuweizi extract could also attenuate mitochondrial Ca^2+^ loading and reduce the sensitivity of hepatic mitochondria to Ca^2+^-dependent MPT caused by carbon tetrachloride [10]. In addition, Wuweizi could enhance both mitochondrial and cellular glutathione levels and antioxidant status by mediating glutathione synthesis and GPx levels, thus reducing ROS and protecting the tissues from oxidative stress in both in vitro and in vivo studies. Although several potential active components of Wuweizi have been reported, a holistic understanding of the molecular mechanisms responsible for their hepatoprotective effects still needs further exploration.

To comprehensively assess herbal pharmacological effects, network pharmacology has been introduced in recent years for exploring the molecular mechanisms of TCMs [11,12]. With a deeply curated network map that describes interactions among molecules, researchers can carry out network-based screening to systematically identify target proteins of herbal medicines and to assess their impacts. Thus, network-based screening appears promising for secondary development of traditional Chinese herbal medicines and mechanisms prediction. Various bioinformatics resources including biological databases and molecular docking software have been developed in recent years, allowing a great opportunity for meeting the demands of rapid systematic screening [13,14,15]. In this study, a network pharmacology study of Wuweizi was established through molecular docking and network analysis based on current identified active components of Wuweizi and potential targets associated with liver diseases. The study may provide a powerful tool for exploring the active mechanisms of TCMs and discovering novel bioactive ingredients of Wuweizi.

## 2. Results and Discussion

### 2.1. Potential Biological Effect of Fructus Schisandrae on Liver Disease Predicted by Network Pharmacological Analysis

**Figure 1:**
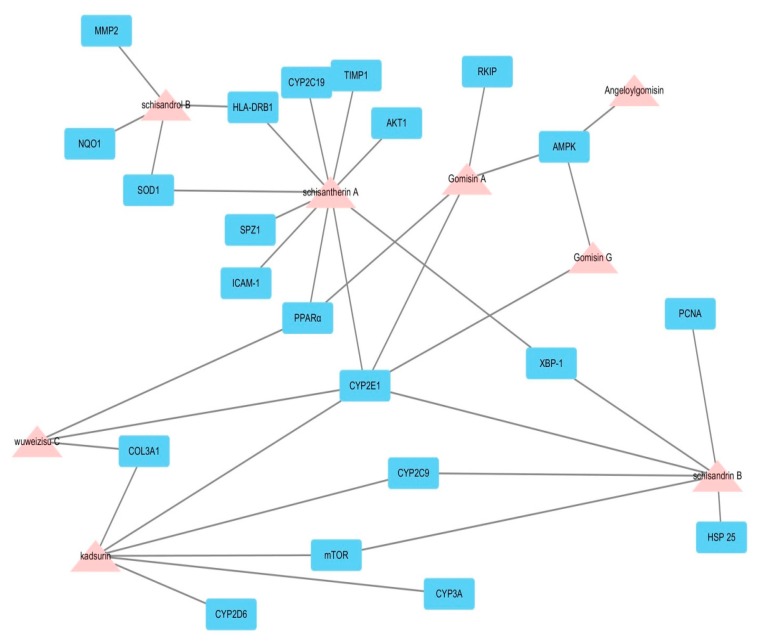
The compound–target network plotting.

In multi-compound medicinal herbs like Wuweizi, many compounds that lack appropriate pharmaceutical properties are believed to fail in reaching the cellular targets; thus, these compounds exhibit limited efficacy that can be neglected. We have identified a total of 117 potentially active chemicals in Wuweizi (Table S1). The targets of these active chemicals of Wuweizi were predicted through molecular docking. To further illuminate the relationship between the effective compounds and potential targets, a compound–target network was built through network analysis. The compound and protein interaction analysis results showed that a total of 21 intracellular targets were predicted to interact with the 8 ingredients of Wuweizi (Figure 1). This network represents a global view of the potential compounds (pink triangles) and targets (blue rectangles) in Wuweizi, and it comprised 29 nodes (8 potential compounds and 21 potential targets) and 46 edges (compound–target interactions). The degree of nodes is a key topological parameter that characterizes the most influential nodes in a network, and we used it to further determine the importance of active components and liver disease targets [16,17]. Those high-degree nodes in the network, which had more compound–target interactions, are likely to play a more important role in liver diseases [16]. Our network analysis results showed that various candidate compounds in Wuweizi were linked to multiple targets, which might exhibit potent hepatoprotective effects. Among the 8 candidate compounds, schisantherin A exhibited the largest number of potential hepatoprotective targets connections (degree = 9), followed by schisandrin B (degree = 6), schisandrol B (degree = 6), kadsurin (degree = 4), wuweizisu C (degree = 4), gomisin A (degree = 4), gomisin G (degree = 2), and angeloylgomisin (degree = 1). For the 21 potential hepatoprotective targets, the network showed CYP2E1 had the largest number of compound–target interactions (schisandrin B, kadsurin, schisantherin A, wuweizisu C, gomisin A, and gomisin G), followed by PPARα (schisantherin A, wuweizisu C, and gomisin A), and AMPK (angeloylgomisin, gomisin A, and gomisin G). The remaining 18 targets showed interactions with only one or two compounds. The information of 21 potential hepatoprotective targets in Wuweizi can be found in Table 1, where all the data were manually collected and integrated from the STITCH, TTD, PharmGKB, and CTD databases. For the 8 potential active chemicals in Wuweizi, some of them have been confirmed by experimental studies. For example, schisandrol B and kadsurin can inhibit CYP2E1 in rat primary hepatocytes and oxidize xenobiotics, such as toxins or drugs, and remove them from the body [18]. Schisantherin A treatment can suppress the enzymatic activities of several CYP450 isoforms, which are related to acetaminophen bioactivation, such as CYP2E1, CYP1A2, and CYP3A11, and further decreased the formation of acetaminophen toxic intermediate *N*-acetyl-*p*-benzoquinone imine (NAPQI) in mouse microsomal incubation system. This study demonstrated that schisantherin A exhibited significant protective actions toward acetaminophen-induced liver injury, which was partially associated with the inhibition of CYP-mediated acetaminophen bioactivation [19]. A recent study showed that gomisin A exhibited significant anti-hepatotoxic action by oral application, hypolipidemic (mainly triglyceridemic) and liver protein synthesis-facilitating actions. The enlargement of the liver seen with treatment of gomisin A is the adaptive hypertrophy, which is due to the induction of drug-metabolizing enzymes [20]. In addition, administration of gomisin G to high-fat-diet-induced obese mice decreased liver weight, hepatic triglyceride (TG) accumulation, and cytoplasmic lipid droplets. These findings demonstrate that Gomisin activated the AMPK pathway and ameliorated HFD-induced hepatic steatosis [21]. Wuweizisu C has also been widely studied by previous researches. It could dose-dependently inhibit fatty degeneration and decrease serum triglyceride. These researches suggested that wuweizisu C could be protective on hepatocellular phenomena such as cell necrosis, fatty degeneration, inflammatory cell infiltration, etc., on human hepatitis [22]. Wuweizisu C also exhibited remarkable antihepatotoxic effects in CCl_4_-induced cytotoxicity, indicating that anti-oxidative action plays an important role in the antihepatotoxic activity of wuweizisu C and Wuweizi [23].

The compound–target network related to hepatoprotective mechanism of Wuweizi in liver disease was shown in the network plotting. This network represents a global view of the potential compounds (pink triangles) in Wuweizi and their intracellular targets (blue rectangles) for liver disease. The network comprised 29 nodes (8 potential compounds and 21 potential targets) and 46 edges (compound–target interactions).

### 2.2. Potential Hepatoprotective Molecular Mechanism of Wuweizi

**Figure 2:**
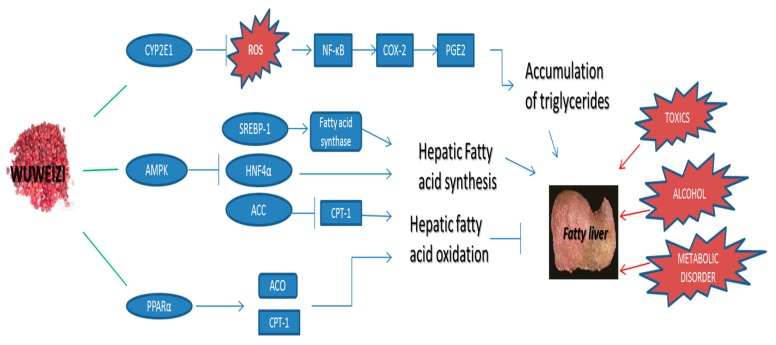
Schematic illustrating proposed potential hepatoprotective mechanisms of Wuweizi.

The potential hepatoprotective mechanisms of Wuweizi were further analyzed by above network pharmacology study. Our results demonstrated that multiple components in Wuweizi could target various intracellular genes associated with liver diseases, especially for fatty liver disease (Figure 2). CYP2E1, the key node in our network plotting, may play a pivotal role in hepatoprotective effects of Wuweizi. Previous studies showed that several risk factors such as alcohol and toxics could induce CYP2E1 activation and further increase hepatic oxidative stress [24]. The reactive oxygen species (ROS) generated by CYP2E1 may lead to the production of reactive aldehydes with potent pro-inflammatory properties [25]. Oxidative stress increases NF-κB activation, thus enhancing the expression of COX-2 and iNOS. The upregulation of COX-2 expression causes the enhanced production of PGE2, which functions through its receptors (EP2 and/or EP4) in hepatocytes to increase the accumulation of triglycerides and further promote the development of fatty liver disease [26,27]. Furthermore, CYP2E1 may be directly associated with the development of drug-induced liver injury during oxidative stress. Inhibiting CYP2E1 activity resulted in a decrease in the production of reactive oxygen species (ROS) during thioacetamide (TAA) [28] or isonicotinyl hydrazine (INH) metabolism [29]. Thus, the regulation of CYP2E1 activity by Wuweizi (schisandrin B, kadsurin, schisantherin A, wuweizisu C, gomisin A, and gomisin G) may inhibit hepatic lipid peroxidation and alleviate hepatic injury.

The network results demonstrated that multiple components in Wuweizi can target various intracellular genes associated with liver disease, especially for fatty liver disease. The CYP2E1, PPARα, and AMPK genes and related pathway may play a pivotal role in the hepatoprotective effects of Wuweizi.

Peroxisome proliferator-activated receptor alpha (PPARα), another key node in our network pharmacology study, can modulate lipid peroxidation in fatty liver disease. PPARα is a kind of nuclear receptor proteins, and important transcription factors in the regulation of some enzymes in the β-oxidation pathway. Previous studies showed that PPARα expression was downregulated in alcoholic liver disease (ALD) and non-alcoholic fatty liver disease (NAFLD) in human liver [30]. Activating PPARα in liver can also prevent acetaminophen-induced liver damage by upregulating mitochondrial glutathione and downregulating fatty acyl-carnitines concentration in blood [31]. In addition, during chronic alcoholic liver injury, PPARα also protects liver tissue by activating fatty acid β-oxidation related pathway [30]. Although PPARα has no direct effects to hepatic lipid metabolism, the transcription level of several PPARα downstream target genes such as acyl-CoA oxidase (ACO) and carnitine palmitoyltransterase-1 (CPT-1) may play a critical role in preventing ALD and NAFLD by regulating mitochondrial and fatty acid β-oxidation [32]. In addition, PPARα can induce a high expression of Acyl-CoA Oxidase (ACO) and carnitine palmitoyltransferase 1 (CPT-1) in adipose tissue and improve glucose homeostasis [33]. Previous studies showed that Wuweizi extract prevented the ethanol-induced decrease in PPARα expression and resulted in a significant decrease in intracellular lipid accumulation in hepatocytes along with a decrease in serum TG levels, and it reversed fatty liver to normal conditions [34]. Our network pharmacology study indicated that the potential active components of Wuweizi such as schisantherin A, wuweizisu C, and gomisin A may exhibit preventive ability for fatty liver disease through modulating the activity of PPARα and regulating the expression of several key genes in mitochondrial and fatty acid β-oxidation [35].

AMP-activated protein kinase (AMPK) may also play a major role in the control of hepatic injury by Wuweizi according to our network results. AMPK integrates nutritional and hormonal signals to promote energy balance by switching on catabolic pathways and switching off ATP-consuming pathways, both by short-term effects on phosphorylation of regulatory proteins and by long-term effects on gene expression. Activating AMPK in liver leads to the stimulation of fatty acid oxidation and inhibition of lipogenesis, glucose production as well as protein synthesis [36]. A previous study showed that enhanced phosphorylation of AMPK can inhibit the accumulation and nuclear translocation of mature sterol regulatory element-binding protein 1 (SREBP-1) and subsequently decreased the mRNA levels of lipogenic genes including acc1, fas, and scd1 [37]. Another study also confirmed that the decreased phosphorylation of AMPK could increase maturation of SREBP-1 and expression of SREBP-responsive genes in the rat liver, and thus inhibit lipid accumulation in rat hepatocytes and human hepatoma cell lines [38]. Activating the phosphorylation levels of AMPK can also inhibit Acetyl-CoA Carboxylase (ACC) and increase CPT-1 expression to reduce hepatic lipid accumulation [39]. In addition, activation of AMPK can reduce the expression of hepatocyte nuclear factor 4α (HNF-4α) and decrease hepatic fatty acid synthesis [40].

Some of the in silico data in our study have been further checked and proved by previous experimental data based on literature research. For example, a recent study results of Western blot indicated that schisandrin B can inhibit cell proliferation by downregulating proliferating cell nuclear antigen (PCNA) and induce cell apoptosis in HCC cells [41]. Incubation with schisandrin B for 6 h caused optimal and dose-dependent increases in cellular 25 kilodalton heat shock proteins (Hsp25) activation at 16 h post-drug exposure in AML12 hepatocytes [42]. Another study found that gomisin A could activate AMP-activated protein kinase (AMPK) and exerts therapeutic effects on liver X receptor (LXR)- or palmitic acid (PA)-induced triglyceride (TG) accumulation in HepG2 cells [21]. These biological results further confirmed the potential prospects of our in silico method in exploring the hepatoprotective mechanisms of Wuweizi.

## 3. Materials and Methods

### 3.1. Database Construction and ADME Screening of Wuweizi Ingredients

All of the known ingredients of Wuweizi were manually collected from related literature and two phytochemical databases: Traditional Chinese Medicine Systems Pharmacology Database (TCMSP, http://ibts.hkbu.edu.hk/LSP/tcmsp.php) and TCM Database@Taiwan (http://tcm.cmu.edu.tw/). An in silico integrative model~ADME was used to select the ingredients with favorable pharmacokinetics properties. The ADME system used in this study included oral bioavailability (PreOB) and Caco-2 permeability (PreCaco-2) prediction. Oral bioavailability (OB) is one of the most vital pharmacokinetic properties of orally administered drugs as it plays an important role for the efficiency of the drug delivery to the systemic circulation [43,44] Here, a reliable in silico screening model (OBioavail 1.1) was employed in OB value calculation of the constituents in Wuweizi. This model was constructed based on 805 structurally diverse drugs and drug-like molecules. Multiple linear regression, partial least square, and support vector machine methods were applied during this model building, ending up with determination coefficient (*R*^2^) = 0.80 and standard error of estimate (*SEE*) = 0.31 for test sets [45,46]. In addition, for orally administered drugs, another pivotal problem is their movement across the intestinal epithelial barrier, which determines the rate and extent of human absorption and ultimately affects its bioavailability [47]. Thus, a preCaco-2 model was used to predict the drug absorption. The phytochemical information of the compounds with their Caco-2 permeability properties were explored using the TCMSP database, the detailed parameters’ information, screening criteria, and calculation can be obtained from TCMSP website (http://ibts.hkbu.edu.hk/LSP/tcmsp.php). Finally, compounds values where OB ≥ 30% and Caco2 ≥ 0.4 cm/s were regarded as active ingredients for further study. It is worth noting that the OB value of schisantherin A and schisandrin B are lower than 30%, but both of them have been widely expected to be the active components in Wuweizi [48,49,50]. Thus, these two compounds were also regarded as bioactive compounds for further analysis.

### 3.2. Preparation of Potential Targets and Ligand Structures

The candidate proteins related to liver diseases were data-mined from literatures and public database sources, including PubMed (www.PubMed.org), PubChem (www.Pubchem.org), DrugBank (http://www.drugbank.ca/), Potential Drug Target Database (http://www.dddc.ac.cn/pdtd/), Therapeutic Targets Database (http://bidd.nus.edu.sg/group/ttd/) and PharmGkb (www.pharmgkb.org). As of today, up to 44 protein targets related to viral hepatitis, fatty liver, liver fibrosis, cirrhosis and liver cancer were obtained (Table 1), and their corresponding X-ray crystallographic structures were directly downloaded from RCSB Protein Data Bank (http://www.pdb.org/).

### 3.3. Molecular Docking and Network Building

All the docking studies were carried out using the protein–ligand complexes with crystal structures. Prior to the docking studies, the protein structures were prepared with Sybyl-X version 2.0 software (Tripos Associates Inc., St. Louis, MO, USA) [51]. The preparation work includes affixing hydrogen atoms and removing co-crystallized ligands as well as water molecules from the protein–ligand complexes. All the amide moieties of glutamine and asparagine in the side chains were regulated to modify their connections with nearby residues and atoms. Then, we used the Surflex-Dock module of Sybyl-X to continue docking. The detailed docking operation process and reliability-validated assay strictly followed the software instruction manual. The targets with docking consensus score greater than 6.0 were selected as the potential targets for further analysis [52,53,54]. The compound–target network was then constructed using Cytoscape 3.0.2 software (http://www.cytoscape.org/) based on the molecular docking score. In the network, nodes stand for compounds and targets, and edges represent the compound–target interactions.

## 4. Conclusions

In this study, a compound–target network was constructed through molecular docking and network analysis. The network predicted the underlying mechanism of hepatoprotective effect of Wuweizi as a therapeutic approach to treat liver diseases, especially for fatty liver disease. This study demonstrated that a network pharmacology-based approach was useful for elucidation of the interrelationship between complex diseases such as liver diseases and Chinese herbal medicines interventions. Therefore, network pharmacology is a powerful tool for exploring the potential action mechanism of TCMs and new active ingredients. However, the current molecular docking results in our network pharmacology studies only provide indeterminate connections between chemicals and corresponding target genes. The accurate action modes between chemicals and target genes such as activation or inhibition are still not clear. To resolve this problem, experimental verification of the potential effective compounds after candidate screening is needed to validate the interactions between drugs and proteins based on theoretical predictions.

## Figures and Tables

**Table 1:** Forty-four protein targets related with viral hepatitis, fatty liver, liver fibrosis, cirrhosis, and liver cancer.

Target Gene	Target Protein	Roles in Hepatoprotective Effects
HLA-DRB1	major histocompatibility complex, class II, DQ beta 1	Anti-virus
IFNL3	interferon lambda 3	Anti-virus
HLA-DRB5	major histocompatibility complex, class II, DQ beta 5	Anti-virus
IL6	Interleukin 6	Anti-virus
NAT2	*N*-acetyltransferase 2	Reduce antituberculosis drug-induced liver injury
STAT3	signal transducer and activator of transcription 3	Regulate hepatic cell growth and apoptosis
CYP2C9	Cytochrome P450 2C9	Regulate drug metabolism
CYP2D6	Cytochrome P450 2D6	Regulate drug metabolism
CYP3A	Cytochrome P450 3A	Regulate drug metabolism
ITPA	inosine triphosphate pyrophosphatase	Regulate drug metabolism
UGT1A1	UDP glucuronosyltransferase family 1 member A1	Regulate drug metabolism
UGT1A3	UDP glucuronosyltransferase family 1 member A3	Regulate drug metabolism
CYP2E1	Cytochrome P450 2E1	Regulate drug metabolism
HSPA1L	Heat shock 70 kDa protein 1L	Regulate drug metabolism
CYP2C19	Cytochrome P450 C19	Regulate drug metabolism
PEMT	phosphatidylethanolamine *N*-methyltransferase	Regulate drug metabolism
PNPLA3	Patatin-like phospholipase domain-containing protein 3	Regulate fatty metabolism
AKT1	v-akt murine thymoma viral oncogene homolog 1	Regulate lipid metabolism
CYP2B6	cytochrome P450, family 2, subfamily B, polypeptide 6	Oxidizes a variety of structurally unrelated compounds, including steroids, fatty acids
CYP1B1	cytochrome P450, family 1, subfamily B, polypeptide 1	Oxidizes a variety of structurally unrelated compounds, including steroids, fatty acids
MMP2	matrix metallopeptidase 2	Tissue repair and induce interstitial fibrosis
PPARα	peroxisome proliferator-activated receptor alpha	Key regulator of lipid metabolism
NFKBIA	nuclear factor of kappa light polypeptide gene enhancer in B-cells inhibitor, alpha	On cellular stimulation by immune and proinflammatory responses
AHSA1	activator of heat shock 90kDa protein ATPase homolog 1	Involve in Grb2-p38 MAPK signaling pathway in fibrosis
NQO1	NAD(P)H dehydrogenase, quinone 1	Involve in alcohol detoxification pathways
HMOX1	heme oxygenase (decycling) 1	Alleviate liver inflammation and reduced oxidative stress
ICAM-1	intercellular adhesion molecule 1	Mediate adhesive interaction in fibrosis process
MAPK1	mitogen-activated protein kinase 1	Regulate cytoskeletal rearrangements in fibrosis process
PRKCB	protein kinase C, beta	Regulate oxidative stress-induced cell damage
ACTA2	actin, alpha 2, smooth muscle, aorta	Involve in myofibroblast cell motility during wound healing in liver
SPZ1	spermatogenic leucine zipper 1	The transcriptional factors of liver fatty acid binding protein
COL1A1	collagen, type I, alpha 1	Transcriptional repressor of the collagen
BCL2	B-cell CLL/lymphoma 2	Regulate the response to mitochondrial damage and related oxidative damage
CCND1	cyclin D1	Functions as a mediator of β-catenin during hepatocarcinogenesis
RKIP	Raf kinase inhibitor protein	Regulate carbon tetrachloride-induced apoptotic hepatic cell death
HERC5	HECT and RLD domain containing E3 ubiquitin protein ligase 5	Acts as a positive regulator of innate antiviral response in liver cells
CDKN1A	cyclin-dependent kinase inhibitor 1A	Regulate hepatic cell cycle in hepatocarcinogenesis
mTOR	mammalian target of rapamycin	Regulate hepatic cell autophagy
EIF6	eukaryotic translation initiation factor 6	Regulate hepatocarcinogenesis by mediating cellular response to DNA damage.
CASP3	caspase 3	Apoptosis inhibitory protein in hepatocarcinogenesis
COL7A1	collagen, type VII, alpha 1	Regulate fibrosis by impacts on extracellular matrix (ECM) proteins such as type IV collagen
COL3A1	collagen, type III, alpha 1	Regulate fibrosis by impacts on extracellular matrix (ECM) proteins such as type IV collagen
HSP 25	25 kilodalton heat shock proteins	Protect cells from oxidation stress
TGFB1	transforming growth factor, beta 1	Regulate liver cancer cells proliferation
TIMP1	TIMP metallopeptidase inhibitor 1	Tissue repair and induce interstitial fibrosis
SOD1	superoxide dismutase 1	Destroys radicals that are normally produced within the cells, such as oxidants
RELA	v-rel reticuloendotheliosis viral oncogene homolog A	Involve in hepatic inflammation
PCNA	Proliferating Cell Nuclear Antigen	Inhibit HCC cell proliferation
XBP-1	X-box-binding protein-1	Protection against endoplasmic reticulum (ER) stress
AMPK	AMP-activated protein kinase	Regulate hepatic fatty acid oxidation

## Supplementary Materials

Supplementary Materials are available online. Table S1: 117 potential active chemicals in Wuweizi.
