# Supplementary material for: A Network Pharmacology-Based Study on the Hepatoprotective Effect of Fructus Schisandrae
Source: Molecules. 2017 Sep 28;22(10):1617. doi: 10.3390/molecules22101617 (PMC6151775; doi:10.3390/molecules22101617)
Supplement: Supplementary file 1 [file molecules-22-01617-s001.pdf]

Table S1. 117 potential active chemicals in Wuweizi.

Molecule Name	MW	OB (%)	Caco-2
(L)-alpha-Terpineol	154.28	48.8	1.39
ZINC02040970	222.41	40.43	1.44
schisantherin A	270.24	9.73	1.57
schisandrin b	400.46	6.25	1.85
(-)-nopinene	136.26	44.84	1.8
calarene	204.39	52.16	1.82
Linalool	170.28	49.37	0.86
beta-Chamigrene	204.39	31.99	1.82
Nootkatin	232.35	31.82	1.1
L-Bornyl acetate	196.32	65.52	1.29
schisandrol B	514.57	31.8	1.33
Moslene	136.26	33.02	1.88
(+)-beta-Phellandrene	136.26	40.3	1.83
()-Cuparene	202.37	38.26	1.88
1H-Cycloprop(e)azulen-7-ol, decahydro-1,1,7-trimethyl-4-methylene-, (1aR-(1aalpha,4aalpha,7beta,7abeta,7balpha))-	220.39	82.33	1.37
Hemo-sol	136.26	39.84	1.83
delta-Terpineol	154.28	55.11	1.28
beta-Gurjunene	204.39	51.36	1.81
(-)-Comphene	136.26	34.98	1.81
beta-Citronellol	156.3	38.89	1.2
[(1S)-endo]-(-)-Borneol	154.28	83.54	1.22
(E)-oct-2-en-4-one	126.22	64.62	1.36
T-Muurolol	222.41	30.41	1.36
()-alpha-Longipinene	204.39	57.47	1.81
Aristolone	218.37	45.31	1.29
3-Furaldehyde	96.09	50.96	1.04
(5S)-5-butyloxolan-2-one	142.22	65.08	1.16
Limetin	206.21	36.63	0.88
beta-Terpinene	136.26	42.29	1.85
(Z)-2-methyl-5-[(1S,2R,4R)-2-methyl-3-methylene-2-norbornanyl]pent-2-en-1-ol	220.39	35.28	1.28
()-Terpinen-4-ol	154.28	81.41	1.36
DBP	278.38	64.54	0.8
β-terpineol	154.28	47.89	1.29
Arnebin 7	272.32	73.85	0.74
beta-Sesquiphellandrene	218.42	30.58	1.82
Thymoquinol	166.24	44.44	1.19
(9aS)-2,5,9,9-tetramethyl-3,4,6,7,8,9a-hexahydrobenzo[7]annulene	204.39	45.36	1.86
(E)-9-Isopropyl-6-methyl-5,9-decadiene-2-one	208.38	60.25	1.45
1,1alpha,4,5,6,7,7alpha,7beta-Octahydro-1,1,7,7alpha-tetramethyl-2H-cyclopropa (alpha)-naphthalen-2-one	218.37	43.91	1.3
1-(1,5-Dimethyl-4-hexenyl)-4-methyl benzene	208.43	30.94	1.83
3alpha-Tigloyloxytropane	223.35	90.82	1
4-Ethenyl-2,2,4-trimethyl-3-(1-methylethenyl)-cyclo-hexane-methanol	222.41	39.67	1.31
Angeloylgomisin O	498.62	31.97	0.83
Schizandrer B	514.62	30.71	0.66
Clupanodonic acid	276.46	44.01	1.22
Epiguaipyridine	215.37	36.98	1.66

Gomisin-A	416.51	30.69	0.63
Gomisin G	508.61	32.68	0.73
Gomisin R	400.46	34.84	0.6
(1R)-1-phenylpropan-1-ol	136.21	70.32	1.26
schizonepetoside A_qt	168.26	60.29	0.87
2-[(1R)-4-methyl-1-cyclohex-3-enyl]propan-2-yl acetate	196.32	33.05	1.37
Thujyalcohol	154.28	73.08	1.2
Wuweizisu C	384.46	46.27	1.08
Wyerone	258.29	79.24	0.77
alpha-Cuparenol	218.37	55.7	1.41
()-beta-Pinene	136.26	44.77	1.85
Terpilene	136.26	33.95	1.84
ACETIC ACID,BORNYL ESTER	196.32	67.15	1.26
(L)-alpha-Terpineol	154.28	48.8	1.39
ZINC02040970	222.41	40.43	1.44
1,8-cineole	154.28	39.73	1.57
(-)-alpha-Pinene	136.26	46.25	1.85
(-)-nopinene	136.26	44.84	1.8
calarene	204.39	52.16	1.82
Linalool	170.28	49.37	0.86
beta-Chamigrene	204.39	31.99	1.82
Nootkatin	232.35	31.82	1.1
L-Bornyl acetate	196.32	65.52	1.29
(R)-linalool	154.28	39.8	1.33
Moslene	136.26	33.02	1.88
(+)-beta-Phellandrene	136.26	40.3	1.83
()-Cuparene	202.37	38.26	1.88
1H-Cycloprop(e)azulen-7-ol, decahydro-1,1,7-trimethyl-4-methylene-, (1aR-(1aalpha,4aalpha,7beta,7abeta,7balpha))-	220.39	82.33	1.37
Hemo-sol	136.26	39.84	1.83
delta-Terpineol	154.28	55.11	1.28
beta-Gurjunene	204.39	51.36	1.81
(-)-Comphene	136.26	34.98	1.81
beta-Citronellol	156.3	38.89	1.2
[(1S)-endo]-(-)-Borneol	154.28	83.54	1.22
(E)-oct-2-en-4-one	126.22	64.62	1.36
T-Muurolol	222.41	30.41	1.36
()-alpha-Longipinene	204.39	57.47	1.81
Aristolone	218.37	45.31	1.29
3-Furaldehyde	96.09	50.96	1.04
(5S)-5-butyloxolan-2-one	142.22	65.08	1.16
Limetin	206.21	36.63	0.88
beta-Terpinene	136.26	42.29	1.85
(Z)-2-methyl-5-[(1S,2R,4R)-2-methyl-3-methylene-2-norbornan yl]pent-2-en-1-ol	220.39	35.28	1.28
()-Terpinen-4-ol	154.28	81.41	1.36
DBP	278.38	64.54	0.8
β-terpineol	154.28	47.89	1.29
Arnebin 7	272.32	73.85	0.74
beta-Sesquiphellandrene	218.42	30.58	1.82
Thymoquinol	166.24	44.44	1.19
(9aS)-2,5,9,9-tetramethyl-3,4,6,7,8,9a-hexahydrobenzo[7]annul ene	204.39	45.36	1.86
(E)-9-Isopropyl-6-methyl-5,9-decadiene-2-one	208.38	60.25	1.45

1,1alpha,4,5,6,7,7alpha,7beta-Octahydro-1,1,7,7alpha-tetramethyl-2H-cyclopropa (alpha)-naphthalen-2-one	218.37	43.91	1.3
1-(1,5-Dimethyl-4-hexenyl)-4-methyl benzene	208.43	30.94	1.83
3alpha-Tigloyloxytropane	223.35	90.82	1
4-Ethenyl-2,2,4-trimethyl-3-(1-methylethenyl)-cyclo-hexane-methanol	222.41	39.67	1.31
Angeloylgomisin O	498.62	31.97	0.83
Schizandrer B	514.62	30.71	0.66
Clupanodonic acid	276.46	44.01	1.22
Epiguaipyridine	215.37	36.98	1.66
Gomisin-A	416.51	30.69	0.63
Gomisin G	508.61	32.68	0.73
Gomisin R	400.46	34.84	0.6
(1R)-1-phenylpropan-1-ol	136.21	70.32	1.26
schizonepetoside A_qt	168.26	60.29	0.87
2-[(1R)-4-methyl-1-cyclohex-3-enyl]propan-2-yl acetate	196.32	33.05	1.37
Thujyalcohol	154.28	73.08	1.2
Wyerone	258.29	79.24	0.77
alpha-Cuparenol	218.37	55.7	1.41
()-beta-Pinene	136.26	44.77	1.85
Terpilene	136.26	33.95	1.84
ACETIC ACID,BORNYL ESTER	196.32	67.15	1.26
